# Functional Transcriptomics of Wild-Caught *Lutzomyia intermedia* Salivary Glands: Identification of a Protective Salivary Protein against *Leishmania braziliensis* Infection

**DOI:** 10.1371/journal.pntd.0002242

**Published:** 2013-05-23

**Authors:** Tatiana R. de Moura, Fabiano Oliveira, Marcia W. Carneiro, José Carlos Miranda, Jorge Clarêncio, Manoel Barral-Netto, Cláudia Brodskyn, Aldina Barral, José M. C. Ribeiro, Jesus G. Valenzuela, Camila I. de Oliveira

**Affiliations:** 1 Centro de Pesquisas Gonçalo Moniz, Fundação Oswaldo Cruz (FIOCRUZ), Salvador, Bahia, Brazil; 2 Vector Molecular Biology Section, Laboratory of Malaria and Vector Research, National Institute of Allergy and Infectious Diseases, National Institutes of Health, Rockville, Maryland, United States of America; 3 Universidade Federal da Bahia, Salvador, Bahia, Brazil; 4 Instituto Nacional de Ciência e Tecnologia de Investigação em Imunologia (iii-INCT), Salvador, Bahia, Brazil; 5 Vector Biology Section, Laboratory of Malaria and Vector Research, National Institute of Allergy and Infectious Diseases, National Institutes of Health, Rockville, Maryland, United States of America; Lancaster University, United Kingdom

## Abstract

**Background:**

*Leishmania* parasites are transmitted in the presence of sand fly saliva. Together with the parasite, the sand fly injects salivary components that change the environment at the feeding site. Mice immunized with *Phlebotomus papatasi* salivary gland (SG) homogenate are protected against *Leishmania major* infection, while immunity to *Lutzomyia intermedia* SG homogenate exacerbated experimental *Leishmania braziliensis* infection. In humans, antibodies to *Lu. intermedia* saliva are associated with risk of acquiring *L. braziliensis* infection. Despite these important findings, there is no information regarding the repertoire of *Lu. intermedia* salivary proteins.

**Methods and Findings:**

A cDNA library from the Salivary Glands (SGs) of wild-caught *Lu. intermedia* was constructed, sequenced, and complemented by a proteomic approach based on 1D SDS PAGE and mass/mass spectrometry to validate the transcripts present in this cDNA library. We identified the most abundant transcripts and proteins reported in other sand fly species as well as novel proteins such as neurotoxin-like proteins, peptides with ML domain, and three small peptides found so far only in this sand fly species. DNA plasmids coding for ten selected transcripts were constructed and used to immunize BALB/c mice to study their immunogenicity. Plasmid Linb-11—coding for a 4.5-kDa protein—induced a cellular immune response and conferred protection against *L. braziliensis* infection. This protection correlated with a decreased parasite load and an increased frequency of IFN-γ-producing cells.

**Conclusions:**

We identified the most abundant and novel proteins present in the SGs of *Lu. intermedia*, a vector of cutaneous leishmaniasis in the Americas. We also show for the first time that immunity to a single salivary protein from *Lu. intermedia* can protect against cutaneous leishmaniasis caused by *L. braziliensis*.

## Author Summary

Sand fly saliva contains potent, biologically active proteins that allow the insect to stop host responses to acquire a blood meal. After repeated exposures, a number of these salivary proteins also induce a response in the host such as antibody production and/or cellular-mediated immunity. In animal models, these immune responses affect *Leishmania* infection. On one hand, immunity to *Phlebotomus papatasi* saliva protected animals against cutaneous leishmaniasis, while on the other hand, immunity to *Lutzomyia intermedia* saliva did not protect but exacerbated this disease. These differences are probably due to the types of proteins present in the saliva of these different sand fly species. The present work focused on isolation and identification of the secreted proteins present in the salivary glands of *Lu. intermedia*, an important vector of *L. braziliensis*, the agent of mucocutaneous leishmaniasis. Saliva from this sand fly contains a number of proteins not present in *P. papatasi* saliva and, with some exceptions; proteins that are homologous between the two species are very divergent. Furthermore, we identified one protein that, after vaccination, induced a cellular immune response able to protect mice against *Leishmania braziliensis* infection. This is the first evidence that a single salivary protein from *Lu. intermedia* can protect mice against this cutaneous leishmaniasis.

## Introduction

Protozoan parasites of the genus *Leishmania* cause a broad spectrum of diseases, collectively known as leishmaniasis, that occur predominantly in tropical and subtropical regions. The sand fly vector delivers the *Leishmania* parasite while acquiring a blood meal, and during this process, the sand fly injects saliva into the host's skin. Salivary proteins have pharmacologic activities that assist in acquisition of a blood meal [1] and, in parallel, these proteins also modulate the function of cells of the immune system [2], [3], [4], [5]. Mice are protected when immunized with bites from *Phlebotomus papatasi* [6] or with plasmid DNA encoding salivary proteins from *P. papatasi* [7] or from *Lutzomyia longipalpis* [8] suggesting that salivary molecules can be envisaged as components of a vaccine against leishmaniasis [9].

Because the composition of salivary molecules varies among distinct sand fly species, it is important to investigate whether the concept of vector-based vaccines can be extended to other *Leishmania* species such as *L. braziliensis*. Of note, American Cutaneous Leishmaniasis, caused by *L. braziliensis*, is distinguished from other leishmaniases by its chronicity, latency and tendency to metastasize in the human host leading to muco-cutaneous leishmaniasis [10]. Surprisingly, immunization with *Lutzomyia intermedia* SGH did not protect mice against *L. braziliensis* infection [11]. An association between the presence of antibodies to *Lu. intermedia* salivary proteins and active disease was reported, suggesting that a humoral response to *Lu. intermedia* SGH may favor *L. braziliensis* infection [11].

Although the salivary gland (SG) transcriptomes of various sand fly species, including *Lu. longipalpis* [12], have been well documented, information regarding the repertoire of *Lu. intermedia* salivary molecules is lacking. The outcome of *Leishmania* infection in mice immunized with *Lu. intermedia* SGH (disease) [11] compared to *P. papatasi* SGH (protection) [13] is distinct. We then hypothesized that such discrepancies could be due to difference in the repertoire of salivary proteins or the difference in the sequences of their salivary proteins. We took the opportunity to characterize the transcriptome from the salivary glands (SGs) of *Lu. intermedia*, the main vector of *L. braziliensis* in Brazil. We also examined the immunogenic properties of a group of salivary proteins and identified one component that inhibited the development of cutaneous leishmaniasis caused by *L. braziliensis* in mice.

## Methods

### Sand flies and preparation of SGH

Adult *Lu. intermedia* sand flies were captured in Corte de Pedra, Bahia. Sand flies were morphologically identified according to the identification key proposed by Young and Duncan. SGs were dissected and stored in groups of 20 pairs in 20 µl NaCl (150 mM)-Hepes buffer (10 mM; pH7.4) at −70°C. Immediately before use, SGs were disrupted by ultrasonication in 1.5-ml conical tubes. Tubes were centrifuged at 10,000×g for two minutes, and the resultant supernatant—SGH—was used for the studies. The level of lipopolysaccharide (LPS) contamination of SGH preparations was determined using a commercially available LAL chromogenic kit (QCL-1000; Lonza Biologics, Portsmouth, NH, USA); LPS concentration was <0.1 ng/ml.

### SG cDNA library

*Lu. intermedia* SG mRNA was isolated from 50 SG pairs using the Micro-FastTrack mRNA isolation kit (Invitrogen, San Diego, CA, USA). The PCR-based cDNA library was made following the instructions for the SMART cDNA library construction kit (BD-Clontech, Mountain View, CA, USA) with some modifications [14]. The obtained cDNA libraries (large, medium, and small sizes) were plated by infecting log phase XL1-blue cells (Clontech, Palo Alto, CA, USA), and the number of recombinants was determined by PCR using vector primers flanking the inserted cDNA and visualized on a 1.1% agarose gel with ethidium bromide (1.5 µg/ml).

### DNA sequencing of the *Lu. intermedia* SG cDNA library

*Lu. intermedia* SG cDNA libraries were plated to approximately 200 plaques per plate (150-mm petri dish). The plaques were randomly picked and transferred to a 96-well polypropylene plate (Novagen, Madison, WI, USA) containing 75 µl of water per well. Four microliters of the phage sample were used as a template for a PCR reaction to amplify random cDNAs. The primers used for this reaction were sequences from the triplEX2 vector. PT2F1 (5′-AAG TAC TCT AGC AAT TGT GAG C-3′) is positioned upstream of the cDNA of interest (5′- end), and PT2R1 (5′-CTC TTC GCT ATT ACG CCA GCT G-3′) is positioned downstream of the cDNA of interest (3′ end). Platinum Taq polymerase (Invitrogen) was used for these reactions. Amplification conditions were 1 hold of 75°C for 3 minutes, 1 hold of 94°C for 2 minutes, and 30 cycles of 94°C for one minute, 49°C for one minute, and 72°C for one minute 20 seconds. Amplified products were visualized on a 1.1% agarose gel with ethidium bromide. PCR products were cleaned using the PCR multiscreen filtration system (Millipore, Billerica, MA, USA). Three microliters of the cleaned PCR product were used as a template for a cycle-sequencing reaction using the DTCS labeling kit from Beckman Coulter (Fullerton, CA, USA). The primer used for sequencing, PT2F3 (5′-TCT CGG GAA GCG CGC CAT TGT-3′) is upstream of the inserted cDNA and downstream of the primer PT2F1. Sequencing reaction was performed on a 9700 Thermacycler (Perkin-Elmer, Foster City, CA, USA). Conditions were 75°C for two minutes, 94°C for two minutes, and 30 cycles of 96°C for 20 seconds, 50°C for 10 seconds, and 60°C for four minutes. After cycle sequencing the samples, a cleaning step was done using Excel Pure 96-well UF PCR purification plates (EdgeBiosystems, Gaithersburg, MD, USA). Fluorescently labeled extension products were purified following Applied Biosystems BigDye XTerminator purification protocol and then processed on an ABI 3730xL DNA analyzer (Applied Biosystems, Inc., Foster City, CA).

### Bioinformatics

Bioinformatics analysis was performed as previously described and raw sequence files were analyzed using a customized program [15]. DNA sequences with Phred quality scores lower than 20, including primer and vector sequences, were discarded. Sequences were then grouped into clusters using a customized program based on identity (95% identity) and aligned into contiguous sequences (contigs) using the CAP3 program [16]. Contigs were then analyzed by blastx, blastn, or rpsblast programs and compared to the non-redundant (NR) protein database of the National Center for Biotechnology Information (NCBI), the gene ontology (GO) FASTA subset, and the conserved domains database (CDD) of NCBI, which contains KOG, protein families (Pfam), and simple modular architecture research tool (SMART) databases. The three potential translations of each dataset were submitted to the SignalP server to detect signal peptides. All the analyzed sequences were combined in an Excel spreadsheet and manually verified and annotated. Sequences were aligned using ClustalW (version 1.4) [17]. For Phylogenetic analysis, statistical neighbour-joining (NJ) bootstrap tests of the phylogenies were done with the Mega package [18].

### SDS-PAGE and proteome analysis

*Lu. intermedia* SGH (equivalent to 60 SG pairs) were run on NuPAGE (4–12%), 1 mm thick (Invitrogen) according to manufacturer's instructions. Proteins were visualized by staining with SimplyBlue (Invitrogen). The gel was sliced into 30 individual sections that were de-stained and digested overnight with trypsin at 37°C. Identification of gel-separated proteins was performed on reduced and alkylated trypsin digested samples prepared by standard mass spectrometry protocols as previously described [19] and performed by the Laboratory of Proteomics and Analytical Technologies (NCI-Frederick, Frederick, MD, USA).

### Ethics statement

Female BALB/c mice, 6–8 weeks of age, were obtained from CPqGM/FIOCRUZ animal facility where they were maintained under pathogen-free conditions. All animal work was conducted according to the Guidelines for Animal Experimentation of the Colégio Brasileiro de Experimentação Animal and of the Conselho Nacional de Controle de Experimentação Animal. The local Ethics Committee on Animal Care and Utilization (CEUA) approved all procedures involving animals (CEUA-L06508-CPqGM/FIOCRUZ).

### Construction of *Lu.intermedia* salivary DNA plasmids and immunization of mice

Ten plasmids, Linb-1 (SP13 protein family), Linb-2 (SP13 family of proteins), Linb-7 (SP15-like protein), Linb-8 (SP15-like protein), Linb-11 (SP13 protein family), Linb-15 (C-type lectin family of proteins), Linb-19 (9.6-kDa protein), Linb-22 (C-type lectin family of proteins), Linb-24 (10-kDa protein), and Linb-28 (SP15-like protein)] encoding *Lu. intermedia* salivary gland-secreted proteins were cloned into VR2001-TOPO vector and purified as previously described [20]. To evaluate the immunogenic potential of proteins present in *Lu. intermedia* saliva, BALB/c mice were immunized intradermally in the right ear three times at two-week intervals with 10 µg of control DNA plasmid or DNA plasmids (recombinant)coding for salivary proteins in 10 µl of sterile water. For generation of immune sera, mice were exposed directly to the bites of *Lu. intermedia* sand flies. In this case, before each sand-fly exposure, female sand flies were left overnight without sugar or water and were used the following day. Ten healthy flies were placed in plastic vials, the upper surfaces of which were covered with a fine netting. Mice were anesthetized and a single ear from mice was pressed closely to the meshed surface of vials containing flies, secured by clamps designed for this purpose. Flies were allowed to feed in the dark for a period of 30 minutes. A minimum of five fully blood-fed flies per ear was required for each sensitization. After three exposures, with a two-week interval between each exposure, mice were euthanized for collection of immune sera.

### Analysis of antisaliva antibodies by ELISA

ELISA microplates were coated overnight at 4°C with 50 µl SGH diluted to five pairs of SGs/ml in coating buffer (NaHCO_3_ 0.45 M, Na_2_CO_3_ 0.02 M, pH 9.6). After washing with PBS-Tween, wells were blocked with PBS-Tween plus 5% dried skim milk for one hour at 37°C. Wells were incubated overnight with sera from mice immunized with control or recombinant plasmids obtained two weeks after the last immunization, diluted (1∶50) in PBS-Tween. After further washings, wells were incubated with alkaline phosphatase-conjugated anti-mouse IgG antibody (Promega, Madison, WI, USA) diluted (1∶5000) in PBS-Tween for one hour at 37°C. Following another washing cycle, wells were developed with p-nitrophenylphosphate in sodium carbonate buffer pH9.6 with 1 mg/ml of MgCl_2_. Absorbance was recorded at 405 nm.

### Analysis of inflammatory immune response in the ear dermis

Following three intradermal inoculations with control (wild type) or with recombinant DNA plasmids (Linb-11 or Linb-7) in the right ear dermis, mice were inoculated with *Lu. intermedia* SGH (equivalent to 1 pair of SGs) in the left ear dermis. Twenty-four and forty-eight hours later, challenged ears were removed and fixed in 10% formaldehyde. Following fixation, tissues were processed, embedded in paraffin, and 5-µm sections were stained with hematoxylin and eosin (H & E) and analyzed by light microscopy. For morphometric analyses, inflammatory cells were counted in three fields/section using a 2005 magnification, covering a total area of 710 µm^2^.

### Intradermal challenge with SGH and *L. braziliensis* parasites

Two weeks following the last immunization with control or with recombinant DNA plasmid (Linb-11) in the right ear dermis, mice were challenged in the left ear dermis by inoculation of stationary-phase promastigotes (10^5^ parasites in 10 ul of saline) + SGH (equivalent to 1 pair of SGs). Lesion size was monitored weekly using a digital caliper (Thomas Scientific, Swedesboro, NJ, USA). *L. braziliensis* promastigotes (strain MHOM/BR/01/BA788) [21] were grown in Schneider medium (Sigma, St. Louis, MO, USA) supplemented with 100 U/ml of penicillin, 100 µg/ml of streptomycin, and 10% heat-inactivated fetal calf serum (all from Invitrogen).

### Parasite load estimate

Parasite load was determined using a quantitative limiting dilution assay and analyzed by the ELIDA program [22]. Briefly, infected ears and retromaxillar draining lymph nodes (dLNs) were aseptically excised at two and eight weeks post infection and homogenized in Schneider medium (Sigma). The homogenates were serially diluted in Schneider medium supplemented as before and seeded into 96-well plates containing biphasic blood agar (Novy-Nicolle-McNeal) medium. The number of viable parasites was determined from the highest dilution at which promastigotes could be grown out after up to two weeks of incubation at 25°C.

### Intracellular cytokine detection by flow cytometry

Reagents for staining cell surface markers and intracellular cytokines were purchased from BD Biosciences (San Diego, CA, USA). Measurement of in vitro cytokine production was performed as described elsewhere [21]. dLNs were aseptically excised at two and eight weeks post infection and homogenized in RPMI medium. Cells were resuspended in RPMI supplemented with 2 mM L-glutamine, 100 U/ml of penicillin, 100 µg/ml of streptomycin, 10% fetal calf serum (all from Invitrogen), and 0.05 M 2-mercaptoethanol. Cells were restimulated in the presence of anti-CD3 (10 µg/ml) and anti-CD28 (10 µg/ml) and were later incubated with Brefeldin A (Sigma) (10 µg/ml). Cells were blocked with anti-Fc receptor antibody (2.4G2) and stained with anti-mouse surface CD4 (L3T4) conjugated to FITC and Cy-Chrome. For intracellular staining of cytokines, cells were permeabilized using Cytofix/Cytoperm (BD Biosciences) and incubated with the anti-cytokine antibodies conjugated to PE:IFN-γ (XMG1.2), IL-4 (BVD4-1D11), and IL-10 (JES5-16E3). The isotype controls used were rat IgG2b (A95-1) and rat IgG2a (R35-95). Data were collected and analyzed using CELLQuest software and a FACSort flow cytometer (Becton Dickinson Immunocytometry System; Becton Dickinson and Company, Sunnyvale, CA, USA). The steady-state frequencies of cytokine-positive cells were determined using LN cells from PBS-inoculated mice.

### Statistical analysis

Data are presented as means ± standard error of the mean. The significance of the results was determined by Kruskal-Wallis tests using Prism (Graph Pad Software, Inc., San Diego, CA, USA), and P values<0.05 were considered significant. To evaluate disease burden in mice, ear thickness of mice immunized with control or recombinant plasmids was recorded weekly for each individual mouse. The course of disease for experimental and control mice was plotted individually, and the area under each resulting curve was calculated using Prism (Graph Pad Software). The significance of the results was calculated by Kruskal-Wallis test.

## Results

### Description of the *Lu. intermedia* SG transcriptome

Assembly of 1,395 high-quality transcript sequences from the cDNA library of *Lu. intermedia* SGs led to the identification of 278 contigs including 193 singletons. Annotation of these contigs—based on several database comparisons—indicated that 76% of the transcripts belong to the putative secreted (S) class, 9% to the housekeeping class (H), and 15% to the unknown (U) class (Table 1). The unknown class may derive from the 5′- incomplete mRNAs in the library or transcripts coding for novel proteins. Notably, the S class had on average 17 expressed sequence tags (ESTs) per contig, while the H and U classes had only 1.46 and 1.57 ESTs/contig, respectively, indicating high expression levels of secreted products in this cDNA library (Table 1). Transcripts coding for proteins associated with synthesis machinery, as expected, were the most abundant in the H class (Supplementary Table S1).

**Table 1:** Classification of transcripts originating from the sialotranscriptome of *Lutzomyia intermedia*.

Class	Number of Contigs	Number of ESTs	ESTs/Contig*	% ESTs	% Contigs
(S) Secreted	61	1064	17.44	76.27	21.94
(H) Housekeeping	83	121	1.46	8.67	29.86
(U) Unknown	134	210	1.57	15.05	48.20
Total	278	1395			

* Average of ESTs per contig (mean).

Inspection of S class contigs, deriving from 1,064 ESTs, identified the enzyme apyrase, 5′-nucleotidase, endonuclease, adenosine deaminase, hyaluroniadase, and glucosidase, all of these previously identified in other sand fly transcriptomes [1], [23], [24], [25] [12], [14], [26], [27], [28], [29](Table 2). Transcripts coding for proteins of ubiquitous distribution include members of the C-type lectin and Antigen 5 families. Insect-specific protein families are represented by the families of yellow proteins, D7 proteins, and SP15 proteins. Sand fly-specific families are also represented, including members of the SP13 family of proteins, anti-FactorXa protein (lufaxin), 10-kDa family, 30-kDa family, and 37–46-kDa family (these names were given in the review article [1]. One salivary protein present in *Lu. longipalpis* was deorphanized (is now referred as the 14.2 kDa salivary protein), and three *Lu. intermedia* orphan peptides were identified. Novel protein families—including a highly expressed family of small peptides accounting for nearly 50% of all ESTs—are part of the novelty of the salivary transcriptome of *Lu. intermedia* (Table 2). Additional analysis of these sequences and their clusterization by different degrees of similarity allowed further identification of divergent or novel protein families, some of which are described below in more detail.

**Table 2:** Most abundant secreted proteins from the salivary glands of the sand fly *Lutzomyia intermedia*.

Sequence Name	NCBI Acc Number	SignalP Site	MW	pI	Number of Sequences	Best Match to NR by BLAST or PSI-BLAST	E-Value	Comment
Linb-1	KA660049	20–21	5.46	3.6	231	TIGR00366 family protein	0.45	SP13 family
Linb-11	KA660050	22–23	4.494	4.2	65	Conserved hypothetical protein	37.0	SP13 family
Linb-10	KA660051	19–20	8.545	7.9	57	UBA/TS-N domain protein	0.30	Novel 8-kDa protein
Linb-7	KA660052	20–21	14.19	9.0	56	SL1 protein	1E-041	SP15 family member
Linb-13	KA660053	22–23	28.43	9.3	55	Antigen 5-related protein	1E-126	Antigen 5-related
Linb-8	KA660054	20–21	14.06	9.2	38	SL1 protein	2E-045	SP15 family member
Linb-17	KA660055	25–26	33.54	8.4	33	Lufaxin, *L. longipalpis*	5E-087	Similar to Factor Xa inhibitor
Linb-21	KA660057	18–19	44	8.4	30	Yellow related-protein	1E-152	Yellow salivary protein
Linb-19	KA660056	20–21	9.548	4.6	30	9.6 KDa salivary protein	1E-005	10-kDa family member
Linb-22	KA660058	19–20	16.37	8.5	28	16.6 kDa salivary protein	2E-025	C-type lectin
Linb-26	KA660060	17–18	22.87	10.0	26	29.2 kDa salivary protein	8E-063	30-kDa Phlebotomine
Linb-25	KA660059	22–23	5.464	8.8	26	Separase	16.0	Novel 5-kDa family
Linb-28	KA660061	20–21	13.82	9.1	23	SL1 protein	4E-036	SP15 family member
Linb-24	KA660062	20–21	4.064	3.9	22	10 kDa salivary Protein	21.0	SP13 family
Linb-29	KA660063	17–18	14.66	9.5	19	Protein NPC2 homolog	0.077	ML domain salivary peptide
Linb-15	KA660065	19–20	16.38	8.7	17	C-type lectin	1E-025	C-type lectin
Linb-2	KA660064	20–21	4.74	4.1	17	4.5 kDa salivary protein	0.51	SP13 family
Linb-14	KA660066	19–20	17.65	9.1	16	16.3 kDa salivary protein	1E-027	C-type lectin
Linb-35	KA660068	17–18	35.78	9.4	15	Putative apyrase	1E-126	Apyrase
Linb-9	KA660067	19–20	7.797	9.8	15	ComE operon protein 1-	5.6	Novel 8-kDa protein
Linb-37	KA660070	16–17	15.41	8.5	14	Protein NPC2 homolog	0.060	ML domain salivary peptide
Linb-36	KA660069	22–23	4.454	9.8	14	Hypothetical protein AWRIB429	28.0	SP13 family
Linb-38	KA660071	16–17	12.34	9.4	13	9.6 KDa salivary protein	1E-012	10-kDa family member
Linb-39	KA660072	23–24	4.347	9.1	12	Hypothetical protein PLA107	37.0	Novel 4-kDa protein
Linb-42	KA660073	18–19	26.06	8.0	10	D7 salivary protein	3E-086	D7 salivary protein
Linb-44	KA660075	18–19	10.53	4.9	9	9.6 KDa salivary protein	2E-004	10-kDa family member
Linb-43	KA660074	20–21	5.616	7.6	9	Putative mature peptide toxin	0.003	Salivary toxin-like peptide
Linb-46	KA660077	21–22	42.55	9.3	8	43.7 kDa salivary protein	1E-138	Putative endonuclease
Linb-45	KA660076	22–23	11.08	5.2	8	14.2 kDa salivary protein	1E-007	14.2-Da salivary protein
Linb-48	KA660078	19–20	19.66	5.8	7	16.6 kDa salivary protein	5E-024	C-type lectin
Linb-41	KA660079	25–26	5.757	7.8	6	Tau-theraphotoxin-Pc1b	0.012	Salivary toxin-like peptide
Linb-49	KA660080	20–21	13.37	5.3	6	Surface antigen ariel1	4E-005	Hypothetical secreted
Linb-54	KA660087	19–20	24.65	9.3	5	Putative hyaluronidase	3E-078	hyaluronidase
Linb-55	KA660081	17–18	16.08	9.1	5	Hypothetical protein	0.24	ML domain salivary peptide
Linb-58	KA660086	17–18	16.18	8.7	5	NPC2-like protein	0.44	ML domain salivary peptide
Linb-59	KA660088	20–21	13.94	9.2	5	SL1 protein	6E-036	SP15 family member
Linb-50	KA660082	22–23	5.759	9.2	5	Hypothetical protein	2.0	Novel 6-kDa protein
Linb-23	KA660083	28–29	4.126	3.9	5	Hypothetical protein PNA2_1425	4.3	SP13 family
Linb-60	KA660084	20–21	4.799	7.8	5	Putative mature peptide toxin-like	0.23	Salivary toxin-like protein
Linb-40	KA660085	25–26	6.288	8.3	5	U21-theraphotoxin-Cj1a	8E-005	Salivary toxin-like

#### SP13 protein family

**Figure 1:**
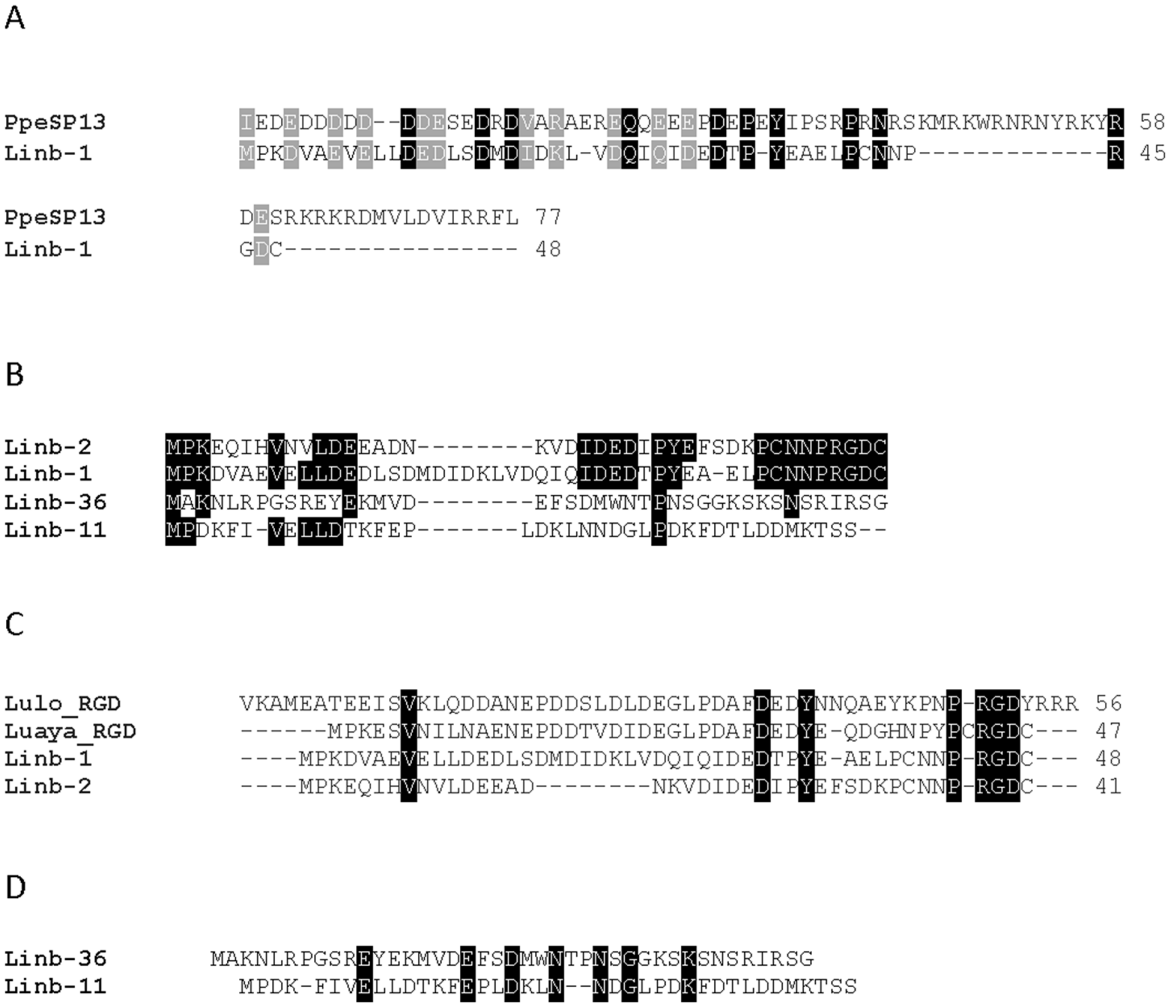
The SP13 protein family of *Lutzomyia intermedia*. A) ClustalW alignment of the deduced protein sequences from *Lu. intermedia* Linb-1 (accession number KA660049) and PpeSp13 (accession number ABA43061.1), a salivary protein *from Phlebotomus perniciosus*. (B) ClustalW alignment of the deduced protein sequences from Linb-1 (accession number KA660049), Linb-2 (accession number KA660064), Linb-11 (accession number KA660050) and Linb-36 (accession number KA660069). (C) ClustalW alignment of the deduced protein sequences from Linb-1, Linb-2, Linb-11 and Linb-36, LuloRGD from *Lu. longipalpis* (accession number AAD32196) and LuayaRGD *from Lu. ayacuchensis* (accession number BAM69127.1). (D) ClustalW alignment of the deduced protein sequences from Linb-11 and Linb-36. Black-shaded residues represent identical amino acids and grey-shaded residues represent similar amino acids.

Six deduced peptide sequences, including Linb-1 (accession number KA660049), Linb-11 (accession number KA660050), Linb-2 (accession number KA660064), and Linb-36 (accession number KA660069) provided weak matches to members of the SP13 family of short (_∼_4.5 kDa) salivary peptides (Table 2) first described in the salivary gland transcriptome from *Phlebotomus perniciosus* [1], [24]. There is 34% identity and 45% similarity between Linb-1 and PerSP13 (Accession number ABA43061.1) from *P. perniciosus* (Figure 1A). This peptide family, which includes Linb-1, assembled from 231 ESTs, and Linb-11, assembled from 65 ESTs, is well expressed in *Lu. intermedia*. Sequence alignment of Linb-1, Linb-11, Linb-2, and Linb-36 shows limited conserved aminoacids (Figure 1B). Importantly, this alignment revealed two groups, one that includes Linb-1 and Linb-2 and that contains a RGD domain at their carboxy terminal end and another group, containing Linb-36 and Linb-11, that does not have this domain (Figure 1B). The Linb-1 and Linb-2 RGD domain is surrounded by cysteine residues, this is typical of platelet aggregation inhibitors of the disintegrin family [30], [31]. Sequence alignment of Linb-1 and Linb-2 with LuloRGD (accession number AAD32196), the salivary protein from *Lu. longipalpis*, which also belongs to the SP13 family of proteins [12] and with LuayaRGD (accession number BAM69127.1), a salivary protein recently described in the transcriptome of *Lu. ayacuchensis* [27] revealed a significant number of conserved amino acids, particularly at the carboxy terminal end (Figure 1C). The second group (Linb-11 and Linb-36) that does not have the RGD shows the presence of conserved amino acids, however, these two sequences did not retrieve any other sequence form the non-redundant database. Linb-11 (but not Linb-36) has a KTS domain in its carboxy terminus but lacks surrounding cysteine residues. The KTS domain, also present in disintegrins, is associated with angiogenesis inhibition [32].

#### *Lu. intermedia* Lufaxin-like family

**Figure 2:**
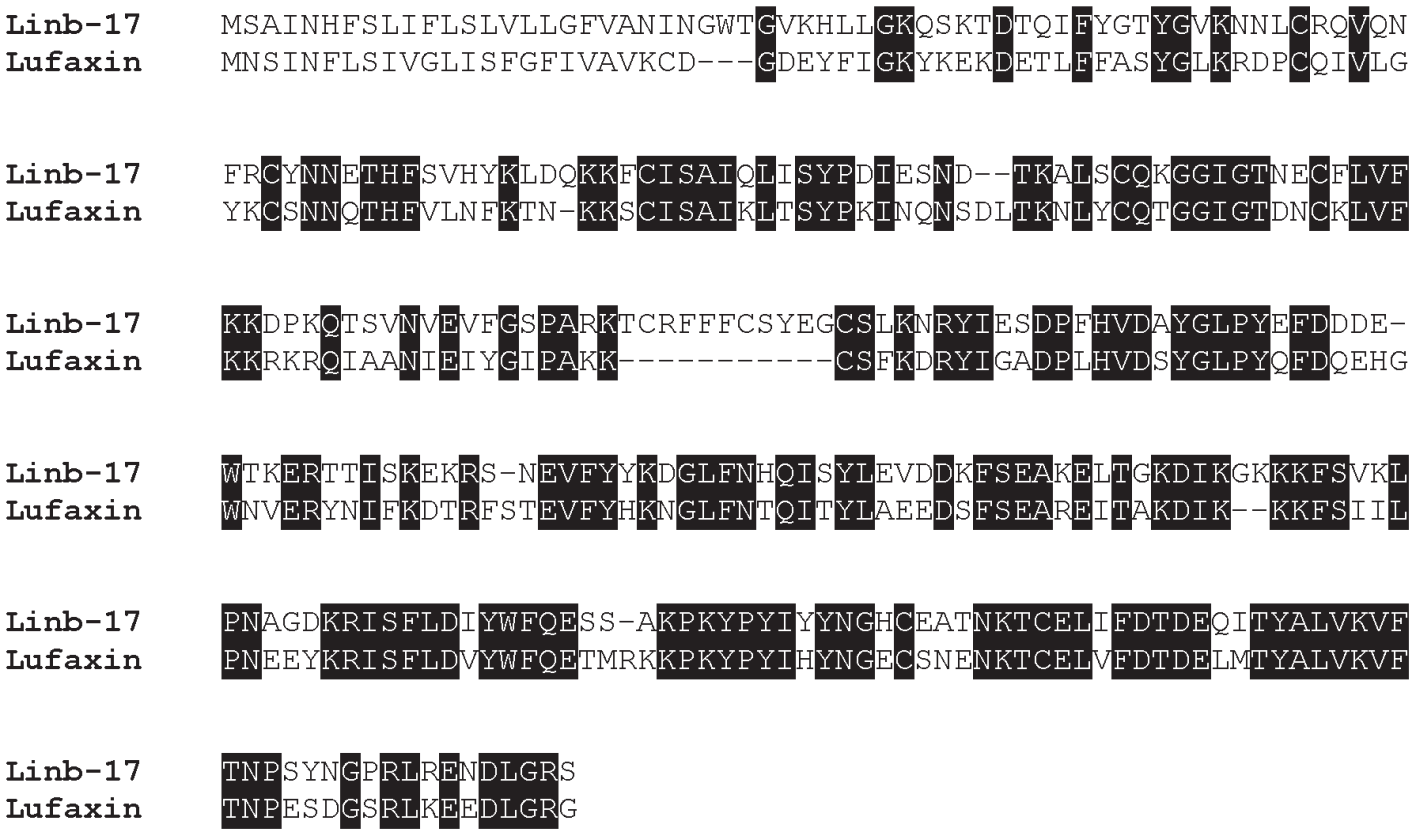
The Lufaxin-like protein family of *Lutzomyia intermedia*. ClustalW alignment of the deduced protein sequences from *Lu. intermedia* Linb-17 (accession number KA660055) and Lufaxin (accession number AAS05319.1), the salivary anticoagulant from *Lu. longipalpis*. Black-shaded amino acids represent identical amino acids.

The salivary anticoagulant from *Lu. longipalpis*, Lufaxin—a specific factor Xa inhibitor—was recently identified and characterized [33]. We also identified a putative Lufaxin-like protein (Linb-17, accession number KA660055) in *Lu.intermedia* that shows a high degree of similarity at the amino-acid level with Lufaxin (accession number AAS05319.1) (Figure 2), suggesting this protein may also have an anti-factor Xa inhibitory activity.

#### *Lu. intermedia* yellow family

**Figure 3:**
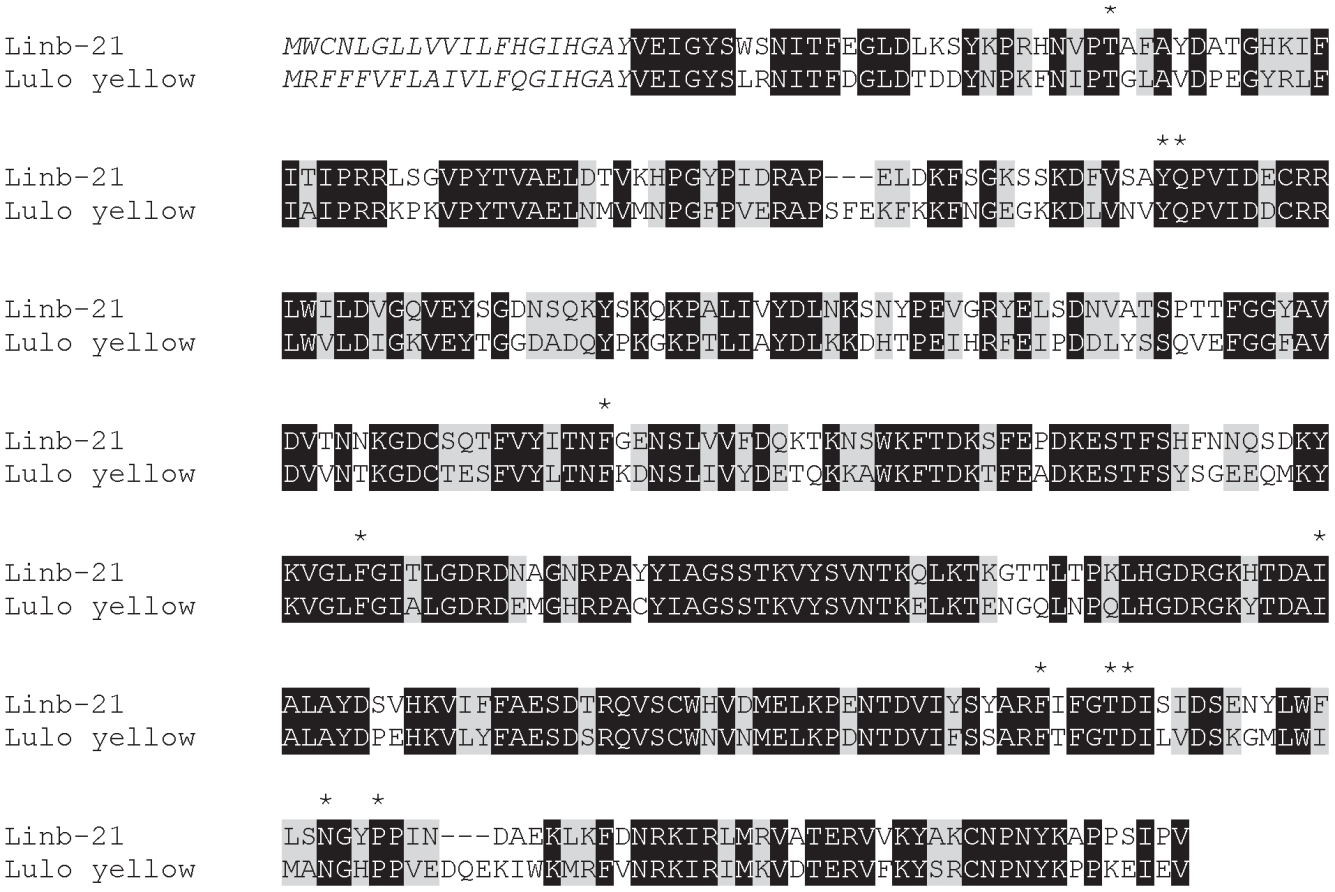
The yellow protein family of *Lutzomyia intermedia*. ClustalW alignment of the deduced protein sequences from *Lu. intermedia* Linb-21 (accession number KA660057) and yellow protein LM17 (accession number AFP99235.1) from *Lu. longipalpis*. Black-shaded amino acids represent identical amino acids, grey-shaded amino acids represent similar amino acids, and amino acids in italics represent the signal secretory peptide. (*) represents amino acids involved in the serotonin binding site for the LJM17 salivary protein from *Lu. longipalpis*. Black-shaded residues represent identical amino acids and grey-shaded residues represent similar amino acids.

The yellow family of proteins is present in the SGs of *Lu. intermedia*. We identified two contigs, one representing a full-length protein (Linb-21, accession number KA660057) with high similarity to the yellow salivary protein LJM17 (Accession number AFP99235.1) from *Lu. longipalpis* (Figure 3) that was recently shown to function as a biogenic amine-binding protein [34]. The essential binding amino acids are highly conserved in the *Lu. intermedia* salivary yellow-related protein (Figure 3). The second contig identified (yellow-related salivary protein) represents a partial protein (Accession number AFP99277.1) with similarities to the *Lu. longipalpis* LJM11 salivary protein (not shown). The yellow proteins from *Lu. longipalpis* (LJM17 andLJM11) are immunogenic in humans and act as markers for *Lu. longipalpis* exposure. Surprisingly, sera from individuals exposed to *Lu. intermedia* bites did not recognize the yellow proteins from *Lu. longipalpis* [35], and this lack of recognition maybe due to some of the differences observed in the amino acid sequence of these two proteins (Figure 3).

#### *Lutzomyia* 10-kDa family

**Figure 4:**
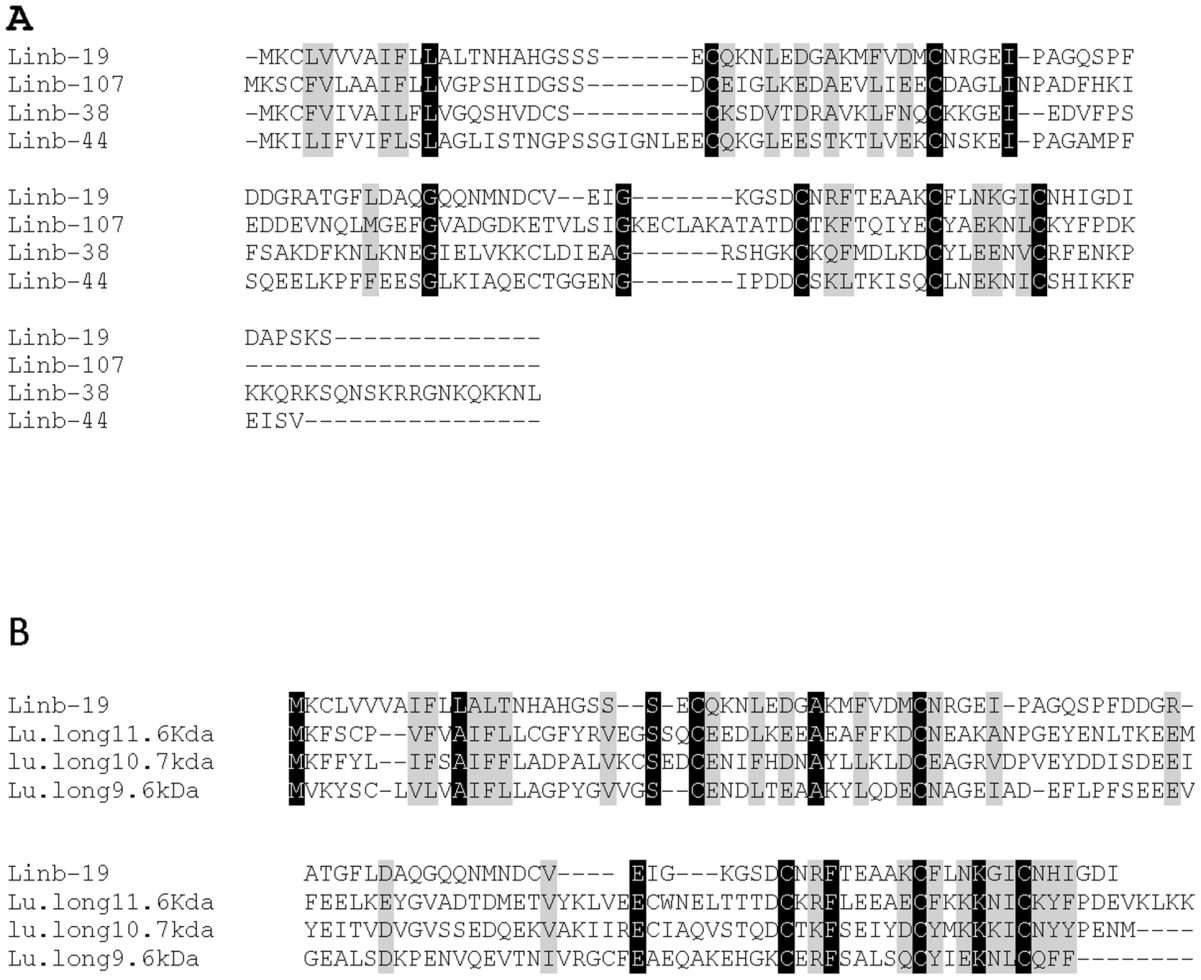
The 10-kDa family of proteins. (A) ClustalW alignment of the deduced protein sequences from *Lutzomyia intermedia* Linb-19 (accession number KA660056) and the 10-kDa members from *Lu. longipalpis*, 11.6 kDa (accession number AAS16912.1), 10.7 kDa (accession number AAR99725.1), and 9.6 kDa (accession number AAR99724.1). (B) ClustalW alignment of the deduced protein sequences from the members of *Lu.intermedia* 10-kDa family of proteins Linb-19 (accession number KA660056) Linb-38 (accession number KA660071), Linb-44 (accession number KA660075) and Linb-107 (accession number JK846100). Black-shaded residues represent identical amino acids and grey-shaded residues represent similar amino acids.

We identified four peptides, Linb-19 (accession number KA660056), Linb-38 (accession number KA660071), Linb-44 (accession number KA660075) and Linb-107 (accession number JK846100) with similarities to the 10-kDa family of proteins [11.6 kDa (accession number AAS16912.1), 10.7 kDa (accession number AAR99725.1), and 9.6 kDa (accession number AAR99724.1)] from *Lu. longipalpis* (Figure 4A). The identified 10-kDa family-like proteins in *Lu. intermedia* are interrelated and they have five highly conserved cysteines thoughtout the molecule (Figure 4B). These peptides may be members of the *Lutzomyia* 10-kDa family that are evolving beyond recognition from their *Lu. longipalpis* homologs.

#### ML domain peptide family

**Figure 5:**
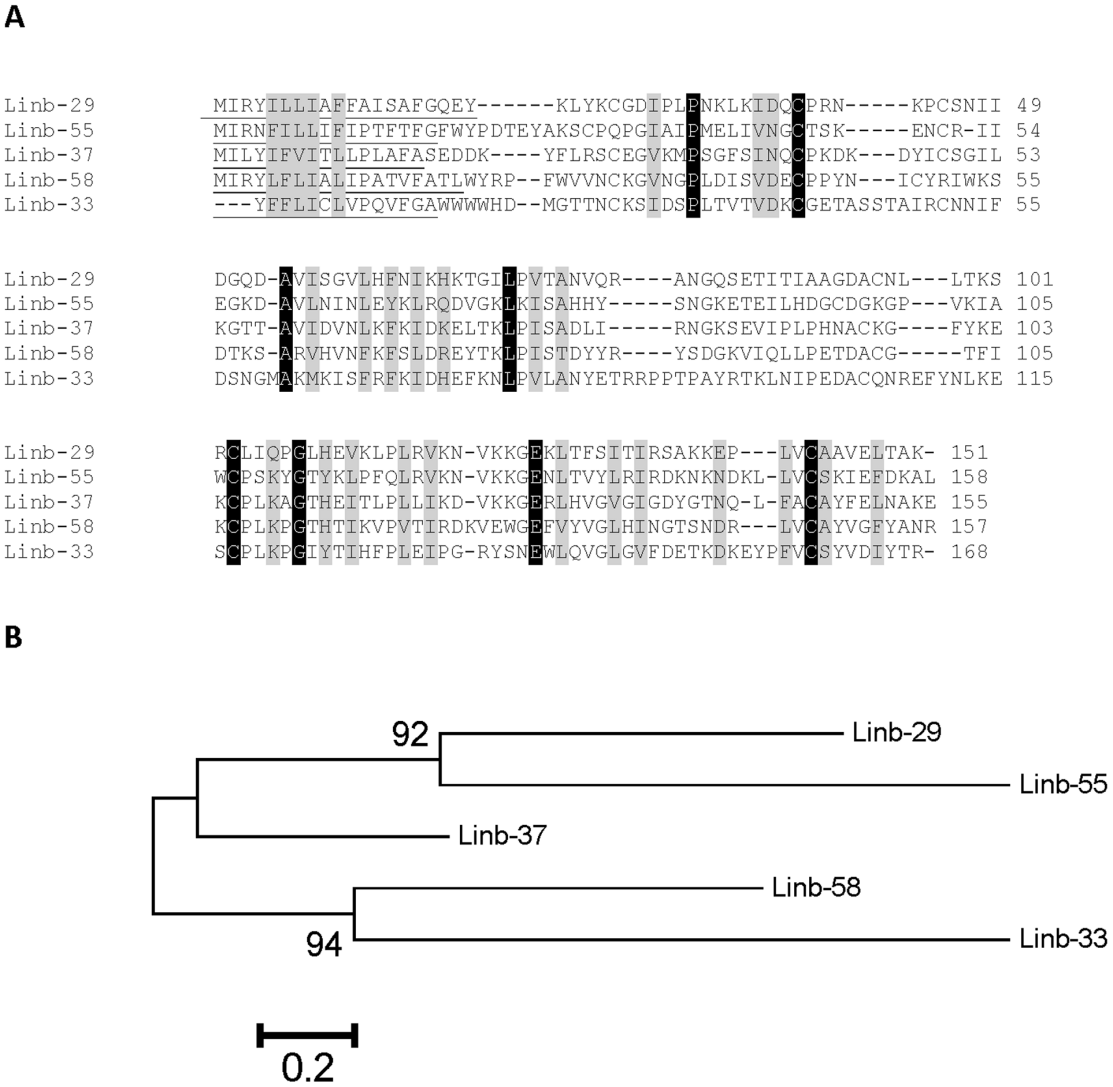
The salivary ML domain protein family of *Lutzomyia intermedia*. (A) ClustalW alignment of the deduced protein sequences from Linb-29 (accession number KA660063), Linb-37 (accession number KA660070), Linb-55 (accession number KA660081), Linb-58 (accession number KA660086) and Linb-33 (accession number JK846303). Black-shaded residues represent identical amino acids and grey-shaded residues represent similar amino acids. (B) Bootstrapped phylogram of the alignment in A. The numbers at the nodes represent the percent bootstrap support. The bar at the basis indicates the amino acid divergence per site.

Five deduced sequences, Linb-29 (accession number KA660063), Linb-37 (accession number KA660070), Linb-55 (accession number KA660081), Linb-58 (accession number KA660086) and Linb-33 (accession number JK846303) had no significant matches to proteins deposited in the NR database of the NCBI but provided matches by rpsblast to the ML domain deposited in the SMART and Pfam databases. The ML domain derives from lipid-binding proteins associated with innate immunity and lipid metabolism. ClustalW alignment of these five sequences (Figure 5A) indicated the existence of 8 identical sites and 35 similar sites for a total of 170 ungapped sites, indicating these proteins result from gene duplications and fast divergence. This is clear from the bootstrapped phylogenetic tree (Figure 5B), indicating these sequences may result from five genes, as each has more than 20% amino acid divergence per site. Transcripts coding for the ML family are relatively well expressed in *Lu. intermedia* SGs; their coding sequences were deduced from 5–19 ESTs, and their relative molecular weight is about 15 kDa. Although The ML family of proteins is relatively common in tick sialomes [36], this family was not previously identified in sand fly transcriptomes.

#### *Lu. intermedia* apyrase family

**Figure 6:**
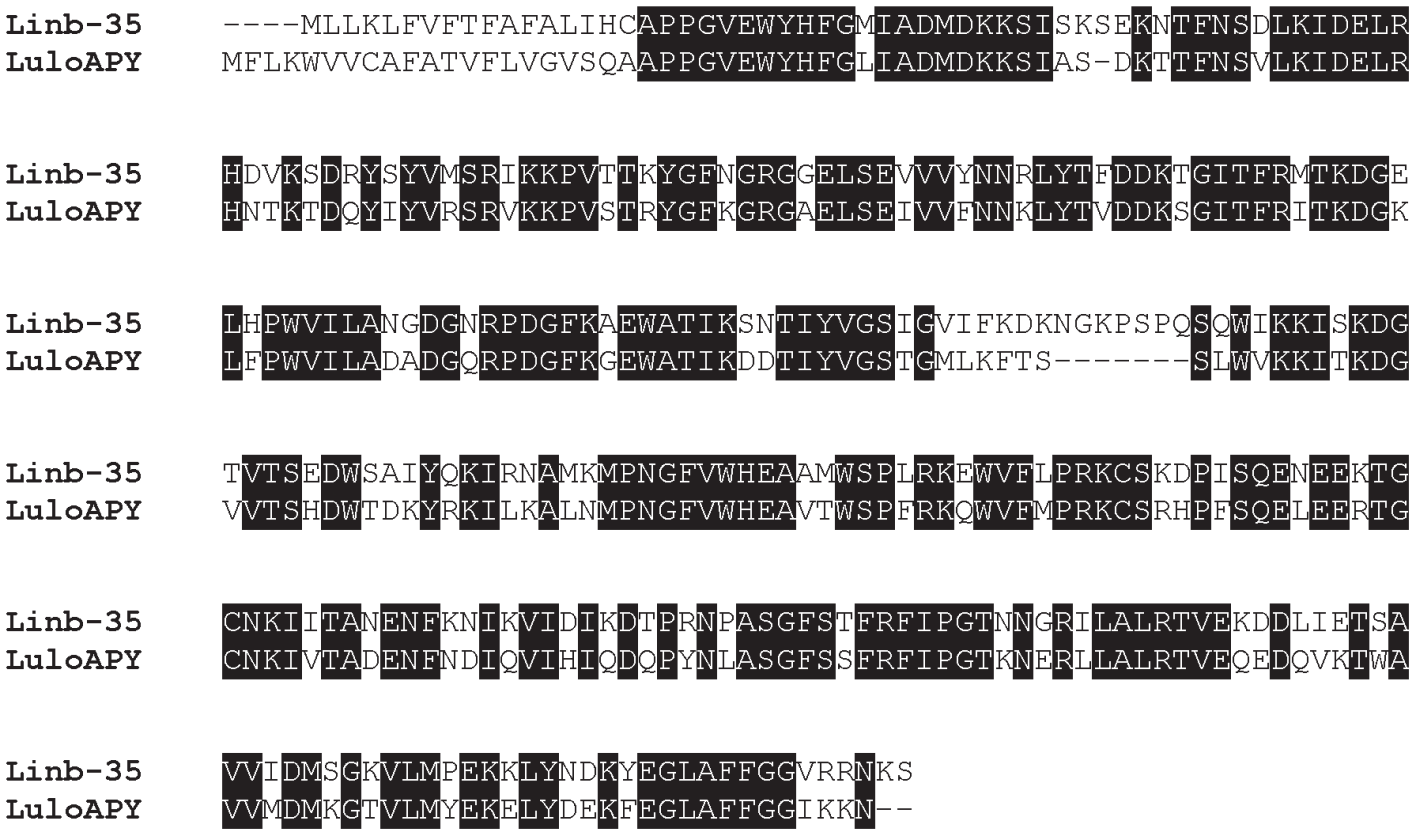
The apyrase from *Lutzomyia intermedia*. ClustalW alignment of the deduced protein sequences from *Lu. intermedia* Linb-35 (accession number KA660068) and the salivary apyrase (LuloAPY) from *Lu. longipalpis* (accession number AAD33513.1). Black-shaded amino acids represent identical amino acids.

The apyrase from sand flies belongs to the Cimex family of apyrases [37] and is very distinct from the 5′ nucleotidase family of proteins found in mosquitoes [38]. We identified a transcript, Linb-35 (accession number KA660068) that showed a significant degree of identity (66% identity, E = 7e-160) with *Lu. longipalpis* salivary apyrase (accession number AAD33513.1) (Figure 6) and with *L. ayacuchensis* salivary apyrase (accession number BAM69098.1) (66% identity, E = 9e-165) (not shown).

#### *Lu. intermedia* toxin-like family

**Figure 7:**
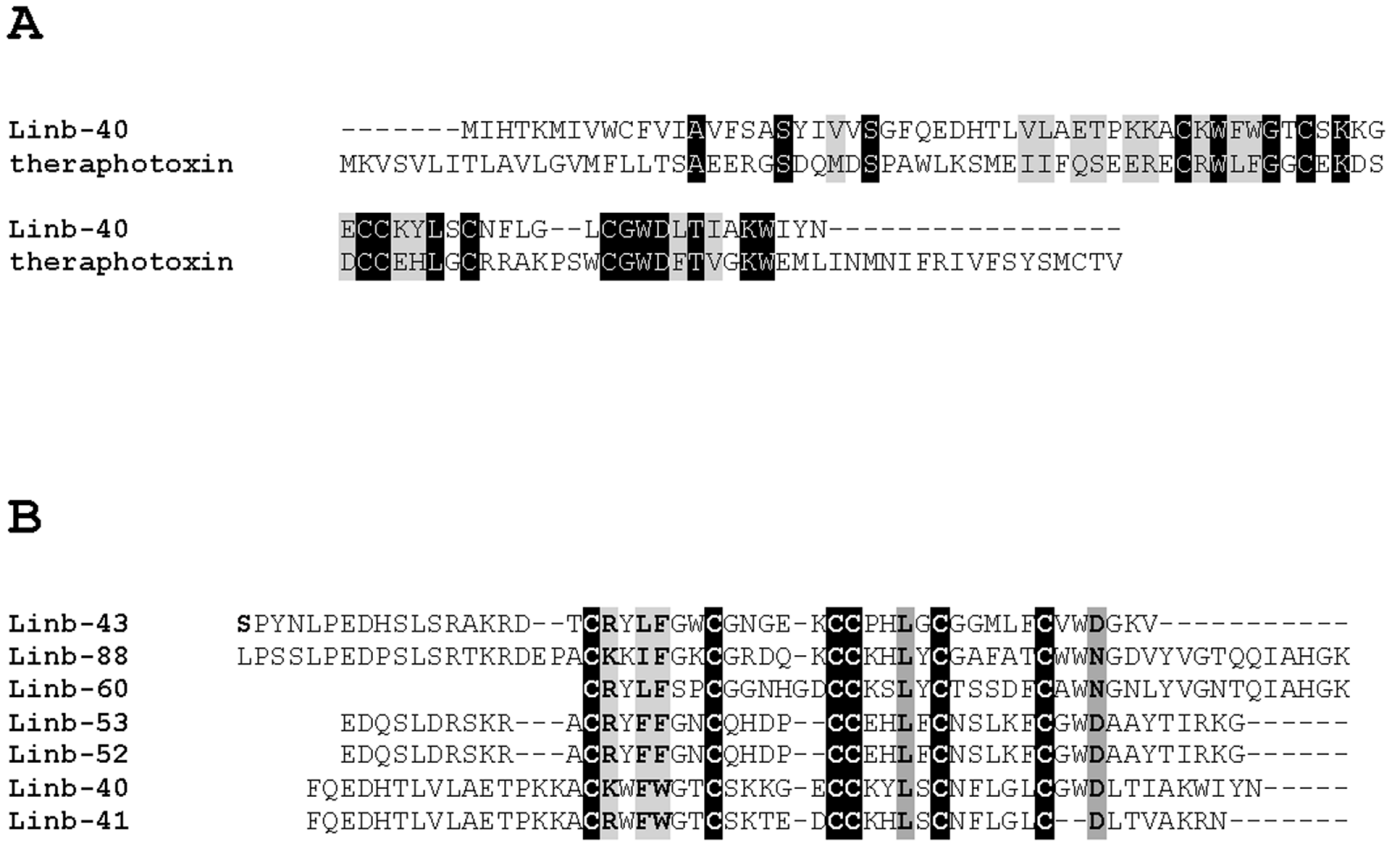
The toxin-like family of *Lutzomyia intermedia*. (A) ClustalW alignment of theraphotoxin from spiders and the salivary Linb-40 (accession number KA660085) from *Lu. intermedia*. (B) ClustalW alignment of *L. intermedia* toxin-like representative members including Linb-40 (accession number KA660085), Linb-41 (accession number KA660079), Linb-43 (accession number KA660074), Linb-60 (accession number KA660084), Linb-52 (accession number KA660090), Linb-53 (accession number KA660094) and Linb-88 (accession number KA660091). Black-shaded residues represent identical amino acids and grey-shaded residues represent similar amino acids.

We identified salivary peptides that match proteins deposited in the NR and Swissprot databases annotated as toxins, such as theraphotoxin (Fig. 7A). Similar proteins have not been identified so far in the salivary transcriptomes of bloodsucking Nematocera. Seven peptides, Linb-40 (accession number KA660085), Linb-41 (accession number KA660079), Linb-43 (accession number KA660074), Linb-60 (accession number KA660084), Linb-52 (accession number KA660090), Linb-53 (accession number KA660094) and Linb-88 (accession number KA660091), deduced from the assembly of 2–9 ESTs, have six conserved cysteine residues including a vicinal doublet in the middle that was identified as the pfam07740 Toxin_12 Ion channel inhibitory toxin from spiders (Figure 7B).

#### Maxadilan-like transcript

**Figure 8:**
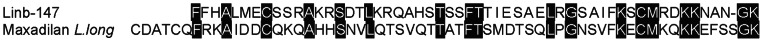
Maxadilan-like protein from *Lutzomyia intermedia*. ClustalW alignment of salivary maxadilan from *Lu. longipalpis* and Linb-147 (accession number JK846521) from *Lu. intermedia* (accession number M77090.1). Black-shaded residues represent identical amino acids.

A single EST, Linb-147 (accession number JK846521) showed a relatively low match to maxadilan (accession number M77090.1) (E value = 1e-04), the salivary vasodilator and immunosuppressive protein present in *Lu. Longipalpis* saliva [39], [40], [41], [42]. This match provided for only 34% identity and 70% similarity over a stretch of 50 amino acids (Figure 8). Interestingly, this transcript is scarcely present in the SG of *Lu. intermedia*, only one transcript was identified in the present cDNA library as compared to 30 transcripts of maxadilan present in the *Lu. longilpalpis* salivary gland transcriptome. It appears that the *Lu. intermedia* gene for maxadilan is evolving beyond recognition from its *Lu. longipalpis* homolog. This is consistent with previous observations on antigenicity and ability of antibodies to block the protein's vasodilatory activity [43] and indicates that the large allelic diversity observed for maxadilan [44] may derive from host immune pressure.

#### Novel peptides with very low similarity to the maxadilan-like peptide

Another four deduced peptide sequences from *Lu. intermedia*, Linb-9 (accession number KA660067), Linb-10 (accession number KA660051), Linb-25 (accession number KA660059) and Linb-50 (accession number KA660082) have borderline 25% similarity among themselves—including the putative maxadilan-like homolog—and lack of significant similarities to peptides deposited in the NR database. Linb-9 and Linb-10 (8-kDa peptide) belong to the same family, and Linb-25 (5 kDa) and Linb-50 (6 kDa) belong to different and independent peptide families. These three families (8 kDa, 6 kDa, and 5 kDa) can be considered novel families, found so far only in *Lu. intermedia*.

### *Lu. intermedia* SG proteome

**Figure 9:**
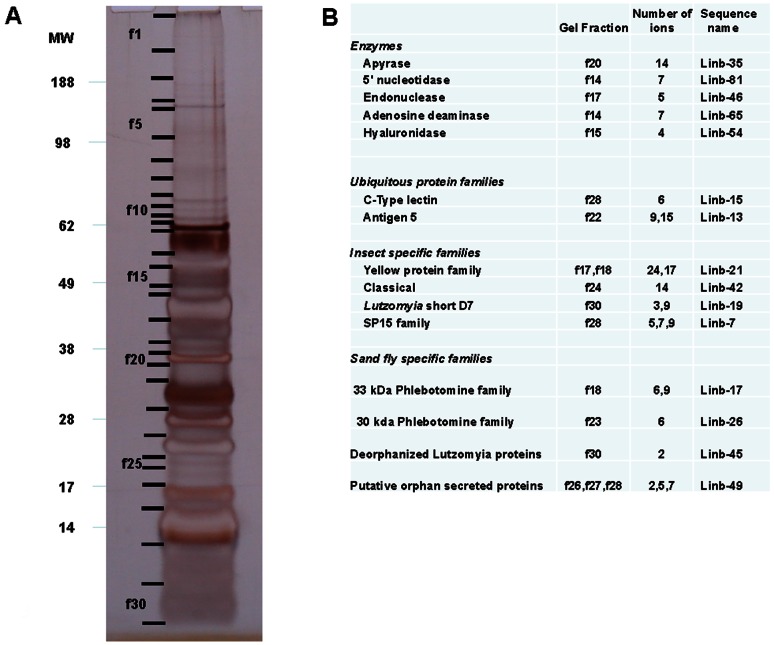
Salivary proteome of *Lutzomyia intermedia*. (A) Separation of *Lu. intermedia* salivary proteins by SDS-PAGE. Indicated fractions (f1–f30) were cut from the gel and submitted to tryptic digestion. Digests were injected on a reverse-phase HPLC column and the eluent directly injected into a mass spectrometer for tandem (MS/MS) identification of peptide sequences, which were compared to the protein database generated by the transcriptome. The numbers on the left side indicate the molecular mass of the standards (MW lane). (B) Identification of the peptides from the fractions selected in part A. Peptide sequences were compared to the *Lu. intermedia* database, and the resulting transcripts were matched with the respective gel fractions. The first column indicates the family of the identified protein, the second column indicates the gel fraction, the third column indicates the number of ions from the gel fraction, and the fourth column indicates the match of the protein with the transcript from the *Lu. intermedia* cDNA library.

We then analyzed the electrophoretic separation of *Lu. intermedia* salivary proteins (Figure 9A), followed by tryptic digestion of selected gel fractions, separation of peptides by reverse-phase HPLC, and subsequent mass spectrometry (Figure 9B). Together with the compiled database of coding sequences, we identified the proteins expressed in the SGs of *Lu. intermedia*. All the fractions displaying a signal from the mass spectrometer matched at least one transcript present in the *Lu. intermedia* cDNA library (Figure 9B). Accordingly, the enzyme apyrase, 5′- nucleotidase, endonuclease, adenosine deaminase, and hyaluronidase were identified at or near the predicted gel migration regions. The proteins Antigen-5, C-type lectin, D7 classical, short, and SP15 were also identified, as were members of the 33- and 30-kDa families of phlebotomines. The deorphanized *Lutzomyia* family member was also identified (Figure 9), as were three members of the putative orphan secreted proteins (not shown on Figure 9). We did not identify the largely expressed SP13 family of short peptides, as they may have migrated out of the gel.

### Immunization with DNA plasmids encoding *Lu. intermedia* salivary proteins induces an immune response in BALB/c mice

**Figure 10:**
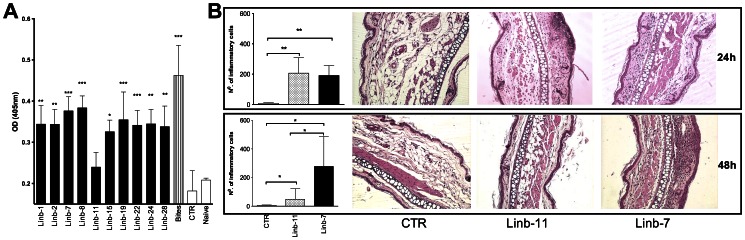
Antisaliva antibody response following immunization with plasmids coding for *Lutzomyia intermedia* salivary proteins. BALB/c mice were immunized with plasmids coding for ten *Lu. intermedia* salivary proteins, wild-type plasmid (CTR) or they were exposed to uninfected sand flies at the right ear. (A) Two weeks after the last immunization, sera were collected and tested by ELISA. (B) Two weeks after the last immunization, mice immunized with Linb-11 or Linb-7 were challenged in the opposite ear with *Lu. intermedia* salivary gland homogenate. Ear sections were obtained at 24 and 48 hours post challenge and stained with H&E. Sections were analyzed by optical microscopy under 100× (bars) or 200× (micrographs) magnification and number of leukocytes enumerated microscopically. Bars represent the mean ± SD of three independent experiments (3–5 mice per group), and the sections are from one representative experiment. Asterisks indicate statistical differences (*, P<0.05; **, P<0.01; ***, P<0.001) comparing experimental to CTR immunized mice.

To identify the immunogenic properties of a group of *Lu. intermedia* salivary proteins, mice were selected randomly and immunized intradermally in the ear with DNA plasmids coding for ten different transcripts identified in this cDNA library: Linb-1 (SP13 protein family), Linb-2 (SP13 family of proteins), Linb-7 (SP15-like protein), Linb-8 (SP15-like protein), Linb-11 (SP13 protein family), Linb-15 (C-type lectin family of proteins), Linb-19 (9.6-kDa protein), Linb-22 (C-type lectin family of proteins), Linb-24 (10-kDa protein), and Linb-28 (SP15-like protein). All recombinant DNA plasmids induced a significant humoral immune response against *Lu. intermedia* SGH when compared with sera obtained from mice immunized with control plasmid or naïve mice (Figure 10A). An exception was the DNA plasmid coding for Linb-11, which induced a low humoral response (Figure 10A). As expected, mice exposed to bites of *Lu. intermedia* sand flies developed a potent humoral response to the salivary proteins of this sand fly (Figure 10A).

A cellular immune response to salivary proteins is associated with protection in animal models of cutaneous leishmaniasis [6], [7]. Therefore, we examined whether Linb-11—a weak inducer of antibody response—could generate a cellular immune response in BALB/c mice. We also tested the response generated by Linb-7, a strong inducer of humoral response in BALB/c mice (Figure 10A). Morphometric analysis of the challenged ears showed a significant increase in cellular recruitment at 24 hours induced by Linb-11 and by Linb-7 (Figure 10B, top panel). Examination of ear sections confirmed this result. At 48 hours, the number of inflammatory cells recruited by *Lu. intermedia* SGH inoculation was significantly lower in Linb-11- compared with Linb-7-immunized mice (Figure 10B), suggesting that Linb-7 leads to a sustained cellular recruitment (Figure 10B).

### Immunization with Linb-11 protects mice against L. braziliensis infection

**Figure 11:**
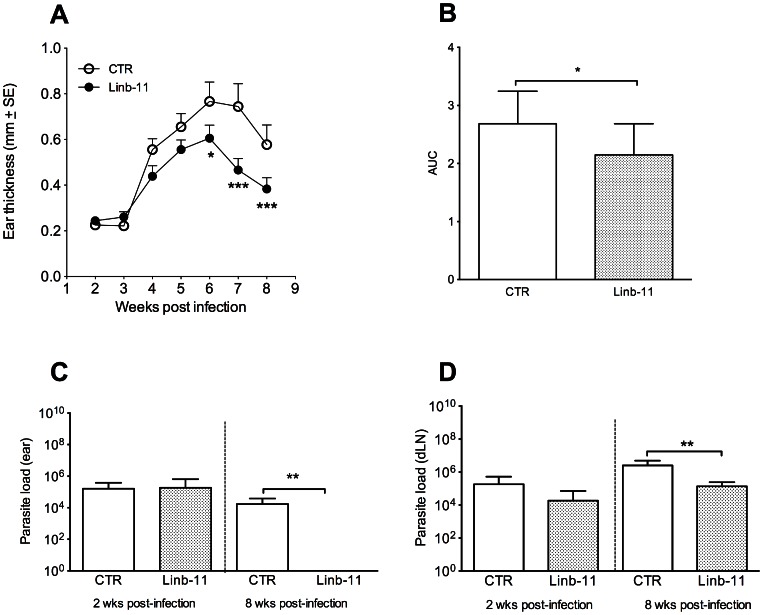
Linb-11 immunization protects mice from *Leishmania braziliensis* infection. BALB/c mice (3–5 per group) were immunized with DNA plasmid coding for Linb-11 or wild-type plasmid (CTR). Two weeks after the last immunization, mice were challenged with *L. braziliensis* plus *Lutzomyia intermedia* SGH in the opposite ear. (A) Lesion development was monitored weekly. (B) Disease burden in indicated by the area under the curve (AUC) for animals vaccinated with CTR or Linb-11. (C) Ear and (D) dLN parasite loads were determined at two and eight weeks post infection via a limiting dilution assay. Data are presented as mean ± SD and are from one experiment representative of three independent experiments. (*, P<0.05; **, P<0.01; ***, P<0.001).

Based on the finding that Linb-11 induces a low humoral immune response and a controlled cellular immune response, we tested whether this protein could protect mice against *L. braziliensis* infection. Linb-11-immunized mice challenged with *L. braziliensis* plus *Lu. intermedia* SGH had significantly smaller lesions (measured by ear thickness) when compared with control mice (Figure 11A). Disease burden, calculated as the area under the curves obtained from Figure 11A (as described in Materials and Methods), was significantly lower following immunization with Linb-11 (Figure 11B). Two weeks post infection, parasite load at the ear (Figure 11C) or in dLN (Figure 11D) were similar in Linb-11-immunized mice versus control mice; however, at eight weeks post challenge, we detected a significant reduction in parasite load in the ear (Figure 11C) and in dLN (Figure 11D) of Linb-11-immunized mice compared with control mice. This decrease in parasite load corroborated the lower ear thickness observed in Linb-11-immunized mice at this same this time point (Figure 11A).

### Linb-11-immunized mice present a rapid expansion of IFN-γ secreting cells

**Figure 12:**
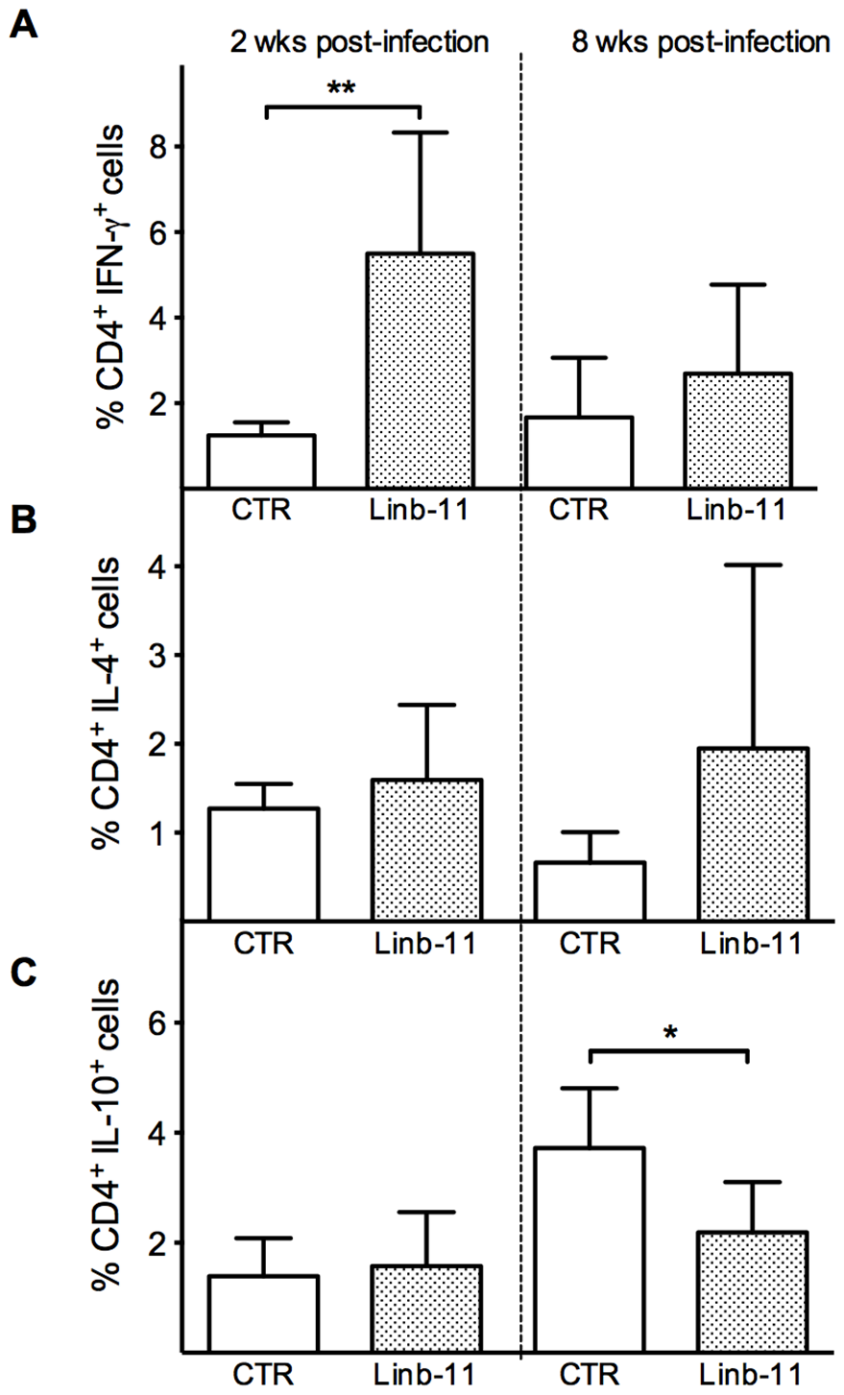
Frequency of cytokine-producing CD4^+^ T cells following immunization with Linb-11. BALB/c mice (3–5 per group) were immunized with DNA plasmid coding for Linb-11 or inoculated wild-type plasmid (CTR). Two and eight weeks after challenge, the percentage of CD4^+^ T cells producing (A) IFN-γ, (B) IL-4, or (C) IL-10 were determined in dLNs following stimulation with anti–CD3 and anti–CD28. The percentage of CD4^+^ cytokine-secreting T cells was determined by flow cytometry. Data are presented as means ± SD and are from one experiment representative of three independent experiments. (*, P<0.05; **, P<0.01).

Evaluation of the frequency of cytokine-secreting cells, at two and eight weeks post challenge with *L. braziliensis* plus *Lu. intermedia* SGH, indicated the presence of higher percentage of CD4^+^ IFN-γ^+^ T cells in Linb-11-immunized mice (Figure 12A). At this same time point, the percentage of CD4^+^IL-4^+^ (Figure 12B) or CD4^+^IL-10^+^ T cells was similar in immunized mice vs. controls (Figure 12C). At eight weeks post infection, the percentages of CD4^+^ IFN-γ^+^ and of CD4^+^ IL-4^+^ T cells were also similar (Figure 12A), whereas the percentage of CD4^+^ IL-10^+^ T cells was significantly lower in Linb-11-immunized mice. We may suggest that in mice immunized with Linb-11, the early (two weeks) predominance of CD4^+^IFN-γ^+^ cells (Figure 12A) results in a better control of lesion development (Figure 11A) and in parasite killing (Figure 11C–D).

## Discussion

Differently from the outcomes observed upon immunization with *P. papatasi* [13] or *Lu. longipalpis* [9] salivary protein, responses to *Lu. intermedia* salivary proteins did not result in protection against *L. braziliensis* infection [11]. We hypothesized that the composition of *Lu. intermedia* salivary proteins could explain these distinct outcomes. In the present work, we characterized the salivary transcripts and proteins present in *Lu. intermedia* SGs and we revealed the presence of *i)* novel sequences of proteins not found in *Lu. longipalpis* or other sand-fly species, including the toxin-like family of proteins, the ML domain containing proteins, and three families of small novel peptides; *ii)* a high degree of divergence between common family of proteins found in *Lu. intermedia* and *Lu. longipalpis*, including maxadilan, the SP13 family of proteins, the 10-kDa family of proteins, and the yellow-related proteins ; and *iii)* an expansion in the SP13, the 9.6-kDa-, the SL1, the C-type, and the ML protein families. Notoriously, the maxadilan homolog in *Lu. intermedia* is very divergent (only 34% identity to maxadilan from *Lu. longipalpis*) and displays low abundance when compared with *Lu. longipalpis* maxadilan. Interestingly, *Lu. intermedia* and *Lutzomyia ayacuchensis*, which lacks salivary maxadilan [27], are vectors of cutaneous leishmaniasis, while *Lu. longipalpis*, the vector for visceral leishmaniasis, has large amounts of maxadilan in the SGs. Moreover, the amount of maxadilan affects the outcome of infection as has been already shown by Warburg et al. [45].

Regarding the *Lu. intermedia* SG transcriptome, the gene expansion of protein families and the sequence divergence of these proteins, compared with homologs found in *Lu. longipalpis*, may explain the distinct immune responses generated by exposure to these two species. Importantly, the divergence is greater when comparing any molecule equal to or smaller than 15 kDa found in the SGs of *Lu. longipalpis* and *Lu. intermedia*. It may be possible that one or more of these divergent molecules in *Lu. intermedia* may generate an immune response that obscures or overrides the immune response of a putative protective protein in the SGs o*f Lu. intermedia*.

We therefore tested whether individual proteins from *Lu. intermedia* could produce a protective immune response different from the one observed when using the whole SGH [11]. We tested ten available DNA plasmids coding for individual *Lu. intermedia* salivary proteins in BALB/c mice and identified one protein, Linb-11, that did not induce an antibody response, produced a transient cellular immune response and protected mice against *L. braziliensis* infection. We then hypothesized that this type of response might modulate disease development, following a live parasite challenge. Indeed, immunization with Linb-11 immunization resulted in lower parasite numbers. This outcome correlated with an early predominance of IFN-γ-secreting cells over IL-4^+^CD4^+^ and IL-10^+^CD4^+^ T cells. These data suggest that CD4^+^IFN-γ^+^ cells may have migrated to the lesion site, leading to macrophage activation, parasite killing and, in turn, smaller lesions. Additionally, the lower percentage of CD4^+^IL-10^+^ cells in Linb-11-immunized mice could also have contributed to the control in lesion development, exerting immunosuppressive functions [46]. We can speculate that the controlled immune response—paralleled by the lack of antibodies—could generate the proper environment to control *L. braziliensis* infection.

It remains to be investigated whether other proteins, such as those initially screened (Figure 10), may display immunomodulatory features similar to Linb-11 or whether other salivary proteins identified in *Lu. intermedia* but not yet tested—or even a combination of proteins—would improve the results obtained. Of note, immunization with plasmids coding for Linb-1 and Linb-2, members of the SP13 protein family, induced a strong humoral response, different from that of Linb-11. This outcome could be explained by the diversity in amino acid sequence of these three molecules. It is also important that candidate molecules, salivary or *Leishmania*-derived, are further tested in the context of natural transmission by infected sand flies. In this stringent scenario, some vaccine candidates have proven effective while others have failed to confer protection [9], [47].

In previous experiments using whole *Lu. intermedia* SGH, we detected a high IL-4:IFN-γ ratio, and this correlated with lack of protection and more severe *Lu. braziliensis* infection [11]. Animals immunized with *Lu intermedia* SGH and challenged with *L. braziliensis* plus SGH showed a significant decrease in CXCL10 expression paralleled by an increase in IL-10 expression [5]. We suggest that when using whole SGH, at least in the *L. braziliensis* experimental model, this salivary mixture overrides priming of the protective immune response induced by Linb-11. In this sense, Oliveira et al. [13] identified in the SGs of *P. papatasi* one molecule, PpSP15, that conferred protection against *L. major* infection, while another protein PpSP44 exacerbated disease. The current results also suggests that *Lu. intermedia* salivary proteins that induce a strong humoral immune response, such as Linb-7, may—to the contrary—exacerbate disease, as seen upon immunization with *Lu. intermedia* SGH [11]. This remains to be tested.

Linb-11 is a small molecule of 4.5 kDa so far found only in sand flies. This molecule belongs to the SP13 family of proteins found in other sand-fly species. *Lu. intermedia* displays an expansion of this protein family by producing six members, as reported in this cDNA library. Nonetheless, Linb-11 induced a mild but protective immune response in an experimental model of infection in the absence of an adjuvant (other than the CpG present in the DNA plasmid backbone). This may suggest that a protein that generates a more controlled immune response may be adequate to limit cutaneous leishmaniasis caused by *Lu. braziliensis*.

## Supporting Information

Table S1 Functional classification of transcripts originating from the sialotranscriptome of *Lutzomyia intermedia* associated with housekeeping function.
(DOC)
