# Supplementary material for: Functional Transcriptomics of Wild-Caught Lutzomyia intermedia Salivary Glands: Identification of a Protective Salivary Protein against Leishmania braziliensis Infection
Source: PLoS Negl Trop Dis. 2013 May 23;7(5):e2242. doi: 10.1371/journal.pntd.0002242 (PMC3662654; doi:10.1371/journal.pntd.0002242)
Supplement: Table S1 — Functional classification of transcripts originating from the sialotranscriptome of Lutzomyia intermedia associated with housekeeping function. (DOC) [file pntd.0002242.s001.doc]

| Table S1: Functional classification of transcripts originating from the sialotranscriptome  of *Lutzomyia intermedia* associated with housekeeping function | | | | | | | | |  |
| --- | --- | --- | --- | --- | --- | --- | --- | --- | --- |
|  |  |  |  |  |  |  |  |  | |
| **Housekeeping Function** | **Number of Contigs** | **Number of ESTs** |  |  |  |  |  |  | |
| Protein synthesis | 41 | 70 |  |  |  |  |  |  | |
| Protein modification | 4 | 4 |  |  |  |  |  |  | |
| Protein export | 2 | 2 |  |  |  |  |  |  | |
| Nuclear regulation | 1 | 1 |  |  |  |  |  |  | |
| Lipid metabolism | 3 | 3 |  |  |  |  |  |  | |
| Energy metabolism | 5 | 5 |  |  |  |  |  |  | |
| Carbohydrate metabolism | 2 | 2 |  |  |  |  |  |  | |
| Amino acid metabolism | 1 | 1 |  |  |  |  |  |  | |
| Extracellular matrix | 2 | 10 |  |  |  |  |  |  | |
| Cytoskeletal | 3 | 3 |  |  |  |  |  |  | |
| Transporters | 4 | 4 |  |  |  |  |  |  | |
| Transcription machinery | 6 | 6 |  |  |  |  |  |  | |
| Signal transduction | 4 | 5 |  |  |  |  |  |  | |
| Unknown conserved | 5 | 5 |  |  |  |  |  |  | |
|  |  |  |  |  |  |  |  |  | |
| Total | 83 | 121 |  |  |  |  |  |  | |
